# A Comparative *In Vitro* Digestion Study of Three Lipid Delivery Systems for Arachidonic and Docosahexaenoic Acids Intended to Be Used for Preterm Infants

**DOI:** 10.3390/molecules29246032

**Published:** 2024-12-21

**Authors:** Blanca Pardo de Donlebún, Assamae Chabni, Celia Bañares, Carlos F. Torres

**Affiliations:** 1 Department of Bioactivity and Food Analysis, Institute of Food Science Research (CIAL, CSIC-UAM), C/Nicolas Cabrera 9, Cantoblanco Campus, Autonomous University of Madrid, 28049 Madrid, Spain; blanca.pardo@csic.es (B.P.d.D.); celia.banares.e@csic.es (C.B.); 2 Department of Production and Characterization of Novel Foods, Institute of Food Science Research (CIAL, CSIC-UAM), C/Nicolas Cabrera 9, Cantoblanco Campus, Autonomous University of Madrid, 28049 Madrid, Spain; assamae.chabni@uam.es

**Keywords:** arachidonic acid, bio-accessibility, docosahexaenoic acid, enzymatic glycerolysis product, formulations, *in vitro* digestion, preterm infants

## Abstract

It is well stablished that docosahexaenoic (DHA) and arachidonic (ARA) acids fulfill relevant biological activities, especially in newborns. However, oils containing these fatty acids are not always optimally digestible. To address this, various formulation strategies and lipid delivery systems have been developed. This study compares the following three formulations in an *in vitro* digestion model to assess bioaccessibility: Enfamil^®^ DHA & ARA (Mead Johnson & Company), an emulsion of Formulaid^TM^, AquaCelle^®^, and pasteurized donated human milk, and a previously characterized enzymatic glycerolysis product (GP) of ARA oil and microalgae oil in a 2:1 (*w*:*w*) ratio. To evaluate digestibility, parameters such as the percentage of oily phase (OP), micellar phase (MP), free fatty acids, and monoacylglycerols in the digestion product (DP) were considered. Additionally, diacylglycerol content in the MP can be used as an indirect marker of the emulsification capacity of the DP, and consequently, as an indicator of bioaccessibility. The GP demonstrated the highest bioaccessibility, with a DP containing more than 80% MP (<14% OP), rich in free fatty acids (60%) and monoacylglycerols (17%). Furthermore, more than 40% of total diacylglycerols were present in MP, highlighting GPs’ potential as a superior delivery system for DHA and ARA in preterm infant formulations.

## 1. Introduction

Human milk is a nutritional reference for the adequate development of breastfed infants regarding the nutritional suitability and efficacy of infant feedings during early life. Docosahexaenoic acid (DHA; 22:6, n-3) and arachidonic acid (ARA; 20:4, n-6), are minor constituents of this secretion and they can be found at different levels and proportions [1]. These concentrations determine the levels of these two relevant fatty acids in infant formulas. Usually, DHA and ARA are found at ∼0.3% and ∼0.6% of total fatty acids, respectively [2]. These two fatty acids have been associated with an adequate visual and cognitive development in newborns [3]. ARA and DHA have also been related to growth and neural development in preterm infants [4]. In addition, these two long-chain polyunsaturated fatty acids (LCPUFAs) are recognized as important modulators of inflammation [5]. Deficient blood levels of these LCPUFAs have been pointed out as risk factors of several neonatal diseases, such as bronchopulmonary dysplasia, retinopathy, and necrotizing enterocolitis [6,7]. The ARA to DHA ratio can also be a useful indicator of adequate neurodevelopment and the risk of bronchopulmonary dysplasia [8]. The metabolism of these two LCPUFAs via cyclooxygenase produces oxylipins, meanwhile lipoxygenase and cytochrome P450 pathways are responsible for producing prostaglandins, thromboxanes, hydroxy- and epoxy-fatty acids, lipoxins, resolvins, protectins, and maresins [9]. Preterm newborns have shown low levels of LCPUFAs [10,11]. This anomaly can be attributed to the shortened gestation, considering that ∼80% of LCPUFAs are transferred to the fetus during the last trimester of pregnancy [12]. In addition, LCPUFAs biosynthesis is deficient in preterm infants, due to the limited ability of its enzymes to transform precursors into these LCPUFAs [13]. Finally, enteral feeds seem inadequate for the efficient absorption of these fatty acids. Therefore, the supplementation of preterm infants with ARA and DHA is a fundamental strategy to alleviate nutritional deficiencies of these fatty acids [14].

Microalgae oil contains a large amount of DHA, which has a low hydrolysis rate when eaten [15]. Therefore, the digestibility and absorption rate of microalgal oil are low, resulting in a low bioavailability of DHA [16]. However, a liposome delivery system, especially containing phospholipids, could improve DHA absorption. The hydrolysis of phospholipids may be the point for DHA release and absorption in the delivery system [2]. Sitosterol is also used to prepare DHA liposomes, which could promote the release of DHA in the small intestine and improve the absorption of DHA [17]. The dietary substrate also affects the release of DHA during digestion.

An important target is the optimization of the bioaccessibility of bioactive fatty acids, preventing excessive lipemia, to guarantee adequate human health. Unfortunately, in addition to the fatty acid composition of edible fats and oils, the complexity of the supramolecular organization of lipids should be also considered. Recently, it has been pointed out that the physicochemistry of lipid structures can condition the hydrolysis of fatty acids and, therefore, their metabolic pathways in the organism. Fatty acid distribution among the different positions in the triacylglycerol or phospholipid molecules, and supramolecular organization, such as emulsions with different sizes, net charges, and crystallization states, modulate digestibility, and therefore, their effect on health. As an example, oils from marine mammals contain TAG in which LCPUFAs are located mainly on sn-1,3 positions. In these cases, a lower level of the hydrolysis of these fatty acids is observed *in vitro* compared to C16:0 and C18:1 fatty acids. Similarly, colipase, needed for pancreatic lipase activity, shows discrimination against DHA, producing an accumulation of DHA in MAG or diacylglycerols (DAGs). This behavior has been attributed to steric hindrance that double bonds close to carboxylic groups produce. Hence, docosapentaenoic acid (DPA), when it is located at sn-1 or sn-3 positions of the glycerol backbone, does not have this hydrolysis resistance [18].

Another factor to take into account is the polymorphism of lipids and its influence on the capacity of lipases to obtain access to and hydrolyze TAG. The physical state and the percentage of solid fat at physiological conditions has not been deeply investigated. The optimization of this physical state could control postprandial lipemia and be a major factor in metabolic diseases [18]. The organization of lipids in food products can modulate their metabolism. Amphiphilic lipids such as phospho- and glycol- and sphingolipids, MAG and DAG, and free fatty acids (FFAs) are organized in various lipid structures such as micelles, vesicles, liposomes, etc. when dispersed in an aqueous medium. FFA hydrolysis by digestive enzymes (lipases and phospholipases) depends on the molecule type in which they are esterified [19].

The impact of emulsified structures on the digestion, absorption, and metabolism of FAs also has to be considered. The dispersibility, the size of the oil droplets, the interfacial activity, and composition may have a crucial influence on the hydrolysis kinetics of lipid digestion and subsequent absorption, as it has been previously investigated [20].

Not only the supramolecular organization of lipids itself, but also the organization of lipids in food matrixes can have enormous influence on their bioavailability. As an example, milk lipids, broadly studied, contain complex milk fat globules in a broad range of particle sizes. These fat globules can be fractionated after milk processing (centrifugation, ultrafiltration, high press homogenization, etc.) [21]. In addition, the milk fat globule is covered by a complex membrane comprising three layers of polar lipids embedding cholesterol, proteins, glycoproteins, enzymes, vitamins, and other minor components. It has been described that this complex structure is highly damaged by mechanical and thermal processing [22].

Different lipid delivery systems have been developed to protect bioactive substances from adverse storage environments and improve their palatability, stability, and bioactivity. Among them are micro- and nanoemulsions, solid lipid nanoparticles, liposomes, and self-emulsifying delivery systems [15,23]. The oral route is the most broadly utilized for the administration of fatty acids [24]. Because fatty acids are not freely dispersed in the aqueous phase, they tend to form large fat globules in the gastrointestinal tract. The enzymatic de-esterification of these lipids occurs at the water–oil interface. Therefore, the smaller the lipid droplets, the faster they hydrolyze and the higher the bioavailability [25]. Of note, the absorption of these lipids is highly dependent on dietary fat, which activates the release of bile acids to emulsify the consumed lipids. Therefore, high intra-individual variation in bioavailability has been a challenge in clinical trials [26]. Different strategies have been developed to reduce lipid droplets in the gastrointestinal tract utilizing different emulsifiers. As an example, polysorbate 80 and polyoxyethylene-polyoxypropylene polymer have been utilized to improve omega-3 ethyl ester bioavailability [27,28].

This study compares three formulations to deliver ARA and DHA in an *in vitro* digestion model to evaluate which formula is the most bioaccessible. One formulation is a human milk in which a commercial mixture of ARA and DHA (Formulaid^TM^ 2:1) was emulsified utilizing Aquacelle^®^ as a self-micro-emulsifying delivery system. The second one is a commercial emulsion (Enfamil^®^ DHA & ARA) from Mead Johnson and the third one is a self-emulsifying lipid delivery system obtained via enzymatic glycerolysis, comprised of mono-, di-, and triacylglycerols of two oils rich in ARA and DHA [11]. The main lipid product used in two of the three *in vitro* digestions (Enfamil^®^ DHA & ARA and human milk emulsion) is Formulaid^TM^ oil (2:1). This product is based on a mixture of triglycerides from fungi and microalgae, containing ARA and DHA in a 2:1 ratio, as well as antioxidants (such as tocopherols). The 2:1 ratio between the fatty acids ARA and DHA present in this product was evaluated in the study by Alshweki et al. [8], where they concluded that this ratio between the two types of fatty acids was the best in terms of the health of premature babies [8]. In addition to this, several studies have been carried out to evaluate the effectiveness and benefits of Formulaid^TM^ oil (2:1) on the health of premature babies, which improved oxidative status despite no change in systemic DHA values [10]. Therefore, this work was to evaluate the *in vitro* digestion of these three lipid formulations, to study how bioaccessible the ARA and DHA fatty acids present in them are, and to establish which one is the best in order to improve the absorption of these fatty acids during premature infants’ digestion process.

## 2. Results and Discussion

### 2.1. In Vitro Gastrointestinal Digestion

A. Formulaid^TM^ emulsion, AquaCelle^®^, and donated breast milk

**Figure 1:**
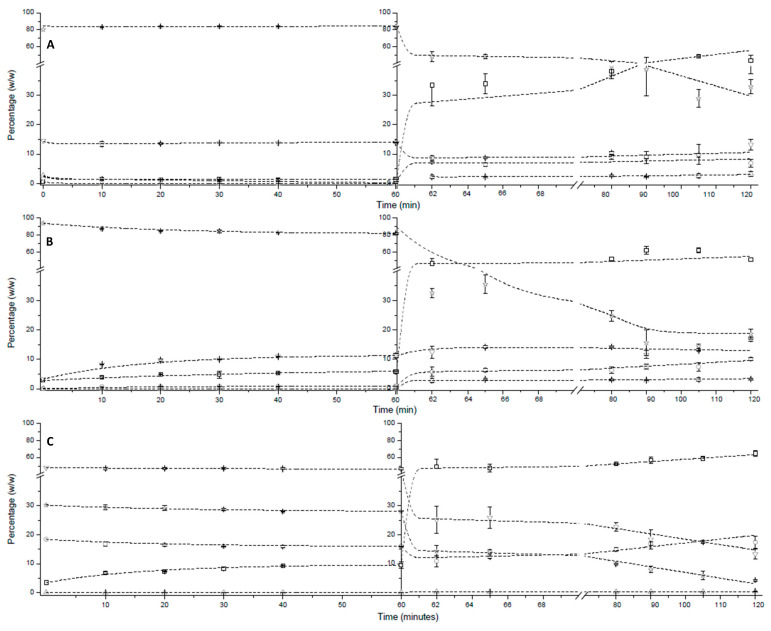
Time course of *in vitro* gastric and intestinal digestion of the emulsion of Formulaid^TM^, AquaCelle^®^, and donated breast milk (**A**), commercial Enfamil^®^ DHA & ARA (**B**), and the glycerolysis product with an ARA to DHA ratio of two to one [11] (**C**). 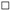 FFA: free fatty acid, 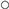 MAG: monoacylglyceride, 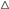 cholesterol, 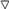 DAG: diacylglyceride, 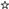 TAG: triacylglyceride.

A gastrointestinal *in vitro* digestion process was carried out. Throughout gastric and intestinal digestion steps, a series of aliquots were taken, and analyzed by gas chromatography. Figure 1A shows the course of gastric and intestinal *in vitro* digestion over time for the emulsion of Formulaid^TM^, AquaCelle^®^, and donated breast milk. In the gastric stage (first 60 min), it is observed that the different lipid species remain almost constant, with minimum variation, over time. These results are similar to those reported in the studies carried out by Menard et al. [29], that consisted in an *in vitro* digestion of an infant formula, in which they did not obtain variation in the different lipid species during the gastric stage. This is due to the fact that gastric lipase has a preference for short- and medium-chain fatty acids, rather than long-chain fatty acids. In addition, gastric lipase is active over a wide pH range (two to eight) and continues to hydrolyze TAG in the intestinal phase [11,30].

Throughout intestinal digestion (60–120 min), two stages are observed: an initial stage, where most of the TAG hydrolysis and formation of digestion products occurred, and a second stage with a slower conversion, probably due to the accumulation of digestion products and lower accessibility of pancreatic lipase. This pattern agrees with the studies of Menard et al. [29], Wang et al. [31], and Martin et al. [32] who also observed a significant increase in free FAs in the first minutes of intestinal digestion *in vitro*.

It is observed that TAGs undergo up to 60% hydrolysis during intestinal digestion, stabilizing at around 35% (*w*/*w*) of the final TAG content. On the other hand, FFAs increase up to almost 55%, followed by MAGs, which increase up to almost 10%. However, the DAGs experience a slight drop of 5% in the first minutes of the stage, and then remain constant over time. Finally, after gastrointestinal digestion, the digestion product (DP) is composed of 55% FFAs, almost 30% TAGs, and the remaining 15% is made up of DAGs, MAGs, and cholesterol. These results point to a poor digestibility of this product compared to the structured TAG that significantly improved the ARA/DHA (*w*/*w*) ratio at the sn-2 position compared to this commercial Formulaid™ formula. Additionally, the DP contained 14.6% TAGs, in contrast to the 35% found in this formula [33]. Also, the results agree with those reported in the studies carried out by C. de Oliveira et al. and Vincent et al. [34,35], in which they verified through *in vitro* and in vivo studies, respectively, that the bioaccessibility of breast milk worsened with pasteurization.

B. Commercial Enfamil^®^ DHA & ARA

Similarly to the previous *in vitro* digestions investigated, the time course of commercial Enfamil^®^ DHA & ARA was studied and the results are shown in Figure 1B. A hydrolysis of TAGs of up to 10% occurs in the gastric stage. In this stage, the digestion product that increases the most is DAG, up to almost 10%. In addition, a slight increase in FFAs is also observed. However, the MAGs remain unchanged during this stage.

In the intestinal stage, a significant hydrolysis of the TAGs occurs, up to 65% (*w*/*w*). It should be noted that, like the previous product (Formulaid^TM^ emulsion, AquaCelle^®^, and donated breast milk), during intestinal digestion two stages are perfectly distinguishable: a first stage in which most of the digestion products are produced, and TAG hydrolysis occurs; and a second stage in which conversion occurs very slowly, probably due to the same reason. On the other hand, FFAs increase by up to 45%, followed by MAGs, which increase by almost 10%. However, DAGs do not undergo any changes at this stage of digestion. Finally, after *in vitro* gastrointestinal digestion, the DP is made up of 50% FFAs and the remaining 50% is made up of TAGs, DAGs, MAGs, and cholesterol. These results show similar digestibility to the Formulaid^TM^, AquaCelle^®^, and donated breast milk formulation. Finally, the phase differentiation after the centrifugation of the digestion product and the distribution of the different lipid classes within these phases will be studied later.

C. Enzymatic glycerolysis product from a mixture of ARA and DHA oils

Figure 1C shows the course of gastric and intestinal digestion over time for the enzymatic glycerolysis product (GP) from a mixture of ARA and DHA oil. In the gastric stage (first 60 min), it can be observed that only a small amount of hydrolysis occurs, releasing ca. 10% of the FAs that comes from the different acylglycerols contained in the GP. On the contrary, during the intestinal phase, ca. 60% of the FFAs was released. The digestion product (DP) contained less than 5% of TAGs and around 15% of DAGs. In addition, the content of MAGs was 16%. These results show a much better digestibility of the GP compared to the other formulations tested in this study. Moreover, they are indicators of a much more adequate digestibility of GP in comparison with the other products previously studied whose *in vitro* digestion is adequate, such as a structured TAG with a high concentration of ARA and DHA at a 2:1 (*w*/*w*) ratio [33] and a predigested mixture produced by the enzymatic esterification of an FA mixture containing ARA and DHA also at a 2:1 ratio [36]. During the gastrointestinal digestion of the structured TAG, its content decreased to 14.6%, along with a reduction in DAGs to 10.1%. Consequently, MAGs and FFAs increased to 19.1% and 50.7%, respectively, whereas the DP of the predigested mixture contained approximately 52% FFAs, 30% MAGs, 3% cholesterol, 12% DAGs, and 3% TAG. It should be noted that although the digestibility of these products is adequate, their production processes are economically unfeasible, unlike the GP.

### 2.2. Hydrolysis Rate

**Figure 2:**
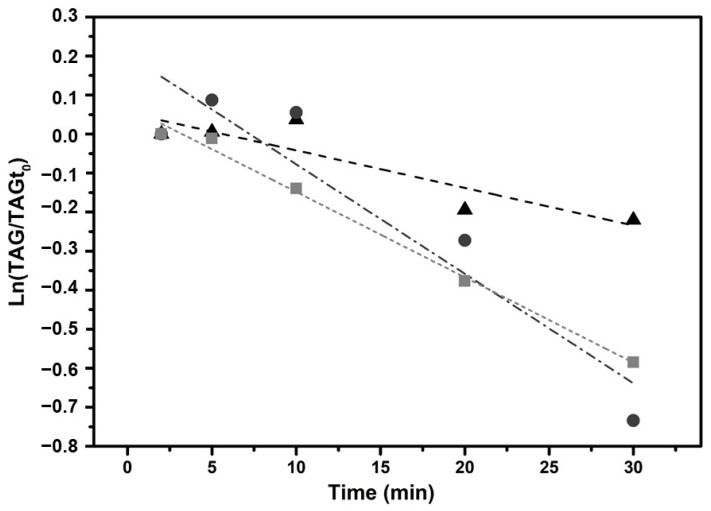
Linear regression of logarithmic total glyceride vs. time for the Formulaid^TM^, AquaCelle^®^, and donated breast milk emulsion (triangles), Enfamil^®^ DHA & ARA (circles), and enzymatic glycerolysis product (squares).

To further investigate the hydrolysis rate of the three formulations, a linear regression of total TAG (%) vs. time is shown in Figure 2. The linearization of this response is carried out by a semi-logarithm representation. Only the first 30 min of the *in vitro* intestinal digestion are plotted in order to avoid data close to the hydrolysis equilibrium. The rate of hydrolysis is obtained from the slope of each line and is listed in Table 1.

The enzymatic glycerolysis product shows a fast and consistent hydrolysis rate with a high correlation of data (R^2^ = 0.992), suggesting a more predictable and stable hydrolysis behavior. In contrast, Enfamil^®^ DHA & ARA has a high hydrolysis rate but with higher variability in the intercept and slope. Formulaid™, AquaCelle^®^, and donated breast milk emulsion, has a slower hydrolysis and moderate correlation, suggesting a less efficient and more variable hydrolysis process. These results are in agreement with those obtained for the *in vitro* digestions of oils and products with PUFA-rich oils [11,37]. The slow hydrolysis rate can be attributed to several factors. One of the main reasons is product inhibition, where the accumulation of non-esterified fatty acids (NEFAs) during *in vitro* digestion can lead to equilibrium between substrate and products, reducing the interaction between pancreatic lipase and substrate [11]. In addition, the saturation of the micellar solubilization of lipolytic products may hinder enzymatic activity, further slowing down hydrolysis. Other factors are the specificity of the enzyme, the distribution of fatty acids in the sn-1, sn-2, and sn-3 positions of triglycerides and the resistance of polyunsaturated fatty acids (such as EPA and DHA) to lipase activity [30,36,37].

### 2.3. Digestion Product (DP)

#### 2.3.1. Formulaid^TM^, AquaCelle^®^, and Donated Breast Milk Emulsion, and Commercial Enfamil^®^ DHA & ARA

**Figure 3:**
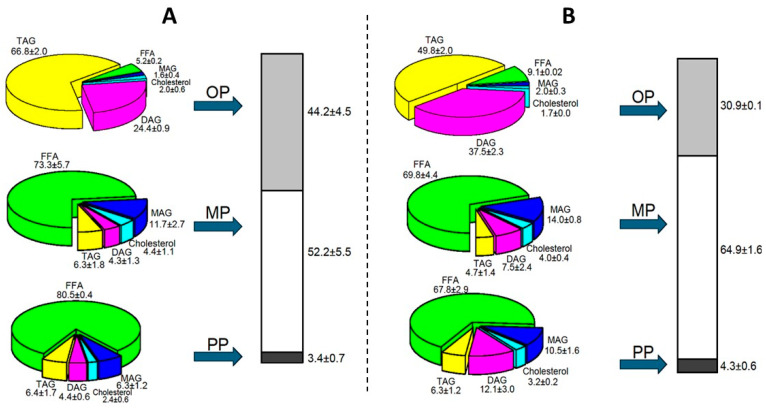
Phase compositions after *in vitro* digestion of the Formulaid^TM^, AquaCelle^®^, and donated breast milk emulsion (**A**), and commercial Enfamil^®^ DHA & ARA (**B**). 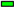 FFA: free fatty acid, 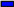 MAG: monoacylglyceride, 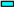 cholesterol, 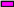 DAG: diacylglyceride, 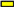 TAG: triacylglyceride. 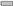 OP: oil phase, 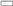 MP: micellar phase, 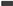 PP: precipitate phase.

Once intestinal digestion was completed, the DP was centrifuged, resulting in three phases being obtained: micellar phase (MP), oil phase (OP), and precipitate phase (PP). Figure 3A shows the percentage of each phase, as well as its composition, obtained after the centrifugation of Formulaid^TM^, AquaCelle^®^, and donated breast milk emulsion DP. It is observed that the OP (44.2%) is very abundant, which in turn is composed mainly of TAGs (66.8%) and DAGs (24.4%). A high percentage of OP implies a larger proportion of undigested lipids after *in vitro* digestion. The MP (52.2%) was mainly composed of FFAs (73.3%) and MAGs (11.7%), and is the bioaccessible fraction. Moreover, only 3.4% of the PP was obtained, formed mostly by FFAs (80.5%) and MAGs (6.3%), insoluble in the micellar phase.

The DP of the commercial formula of Enfamil^®^ DHA & ARA (Figure 3B), after separation by centrifugation, also showed an important OP (30.9%) which points out incomplete digestion and poor bioaccessibility. It should be noted that the MP (64.9%) was more abundant than that observed in Formulaid^TM^, AquaCelle^®^, and donated breast milk emulsion, which shows a little improvement in terms of the digestibility of this emulsion. Again, a very small PP was attained after centrifugation, with a similar composition to the MP.

There results indicated poor digestibility, as the lipids remained unbroken by the digestive enzymes. In the case of oils rich in LCPUFAs, such as ARA and DHA rich oils, a high OP suggests that these oils are less bioaccessible compared to the structured TAG and predigested mixture formed by the enzymatic esterification of an FA mixture of ARA and DHA at a 2:1 ratio, which show a lower OP and better absorption [33,36].

**Figure 4:**
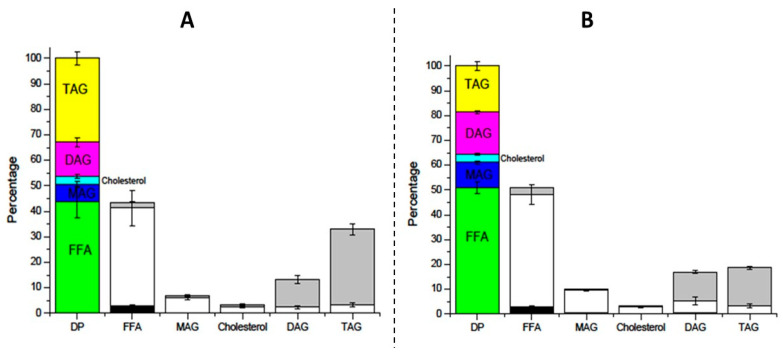
Distribution of each lipid compound among the different phases of digestion product (DP) from the Formulaid^TM^, AquaCelle^®^, and donated breast milk emulsion (**A**), and commercial Enfamil^®^ DHA & ARA (**B**). 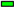 FFA: free fatty acid, 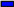 MAG: monoacylglyceride, 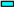 cholesterol, 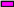 DAG: diacylglyceride, 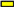 TAG: triacylglyceride. 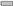 OP: oil phase, 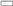 MP: micellar phase, 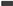 PP: precipitate phase.

Figure 4 shows how the different lipid classes of the DP are distributed within the phases obtained for Formulaid^TM^, AquaCelle^®^, and donated breast milk emulsion (4A), and commercial Enfamil^®^ DHA & ARA (4B), respectively. In the first case, the DP contained less than 10% MAG, which indicates inefficient lipid digestion and reduced bioaccessibility [38]. The commercial emulsion (Figure 4B) shows a slight increase in MAG percentage in the DP that again indicates a little bit better digestibility of this emulsion compared with that of the donated milk emulsion. However, for adequate digestibility, the MAG level should be at least close to 20% [11,30,36]. MAGs are the primary products of TAG breakdown by lipases. When their ratio is low, it suggests an incomplete hydrolysis of fats, which means that fewer lipids are available in a form that can be readily absorbed by the body. This can affect the bioaccessibility of ARA and DHA, and other nutrients, leading to less efficient absorption of nutrients from digested fats [37].

Moreover, it is also important to note that close to 50% of the Formulaid^TM^, AquaCelle^®^, and donated breast milk, DP was composed of DAGs and TAGs, which confirms its poor digestibility and bioaccessibility. It is also possible to see again that FFAs and MAGs are mainly located in the MP, and DAGs and TAGs are found mostly in the OP.

#### 2.3.2. Enzymatic Glycerolysis Product from a Mixture of ARA and DHA Oils

**Figure 5:**
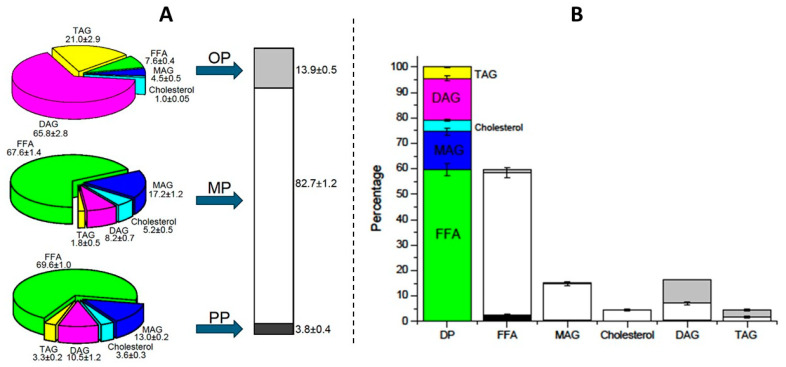
Phase compositions after *in vitro* digestion (**A**) and the distribution of each lipid compound among the different phases (**B**) of enzymatic glycerolysis product DP. 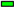 FFA: free fatty acid, 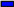 MAG: monoacylglyceride, 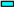 cholesterol, 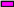 DAG: diacylglyceride, 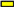 TAG: triacylglyceride. 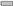 OP: oil phase, 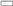 MP: micellar phase, 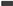 PP: precipitate phase.

Figure 5 shows the percentage of each of the DP phases after the centrifugation of the enzymatic glycerolysis product after intestinal digestion, as well as its composition (A) and the distribution of the different lipid classes in each phase (B). It is observed that the major phase is the MP (82.7%), which is mainly composed of free FAs (67.6%) and MAGs (17.2%). These results are very positive since they indicate ca. 85% of the digested product is bioaccessible for absorption in the small intestine, as explained previously. On the other hand, it is worth highlighting the small amount of OP obtained (13.9%), composed mainly of DAGs (65.8%) and TAGs (21%); so in this case, only a little more than 10% of the initial product is not digested and is not bioaccessible. Finally, only ca. 4% of the PP has been obtained, made up mostly of free FAs (69.6%) and MAGs (13%), insoluble in the MP [11]. These results confirm that the GP has better digestibility and bioaccessibility than the commercial Enfamil^®^ DHA & ARA and Formulaid^TM^, AquaCelle^®^, and donated breast milk emulsion.

Furthermore, Figure 5B indicates that 75% of the DP was composed of FFAs and MAGs. These two lipids were located mainly in the MP. A total of 20% of the digestion product consisted of DAGs and TAGs that were found mainly in the OP. It is important to note that more than 40% of total DAGs are located in the MP, indicating adequate emulsification properties of the DP, which is able to incorporate a significant amount of total DAGs into the MP. For all of these reasons, the GP can be considered as a predigested product and an excellent delivery system for the oral and enteral administration of ARA and DHA, especially crucial in premature newborns.

The use of predigested products [11,36] or structured lipids [33] can be a much more efficient way to deliver ARA and DHA to avoid inadequate pancreatic lipase activity, an inadequate degree of hydrolysis, or emulsification problems, limitations that can be encountered using ARA- and DHA-rich precursor oils or products based on them.

### 2.4. Physical Chemistry Characterization

**Figure 6:**
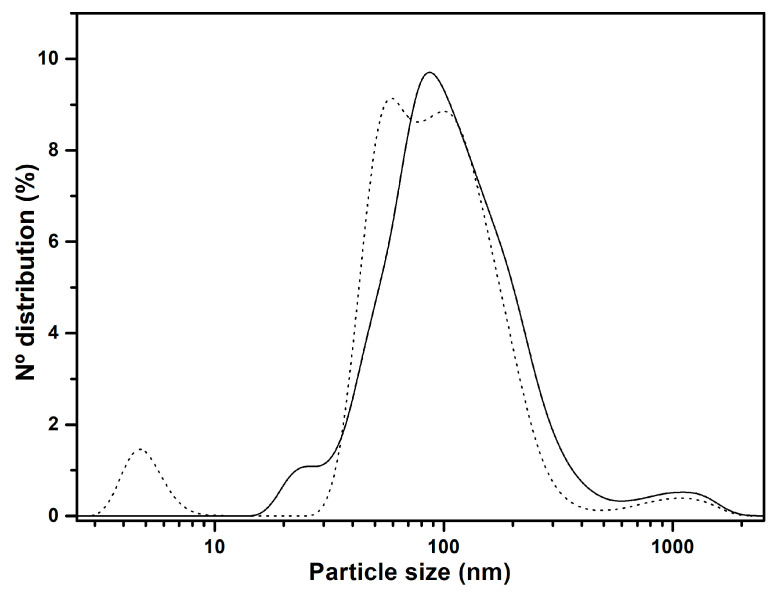
Particle size distribution of the commercial Enfamil^®^ DHA & ARA micellar phase (dots) and glycerolysis product micellar phase (line).

In order to strengthen the bioaccessibility results obtained through the *in vitro* digestion, the physicochemical characterization of two studied products was carried out since they have shown the highest micellar phase (MP); these were the glycerolysis product (GP) and commercial Enfamil^®^ DHA & ARA. The electrical charges (ζ-potential) (Table 2) and particle size (Figure 6) of the MP of each product were determined. Both MPs showed similar and negative high ζ-potential which is indicative of good stability of the microemulsions analyzed. The values of electrical charge can be linked to the presence of anionic species, like dissociated FFAs, which result from lipid hydrolysis during *in vitro* gastrointestinal digestion. Additionally, complex structures formed from bile salts and phospholipids contribute to these values [38,39].

Regarding particle size distribution, in Figure 6 it can be observed that Enfamil^®^ DHA & ARA MP contains a small number of particles around 5 nm that are not detectable in the glycerolysis digestion product. However, in both MPs, the highest number of particles is found around 100 nm, with the GP being slightly more stable. According to certain authors, mixed micelles, or vesicles, are around 100–200 nm in size [39,40]. Mixed micelles with particle size distributions up to 400 nm would be expected to penetrate the mucus layer and consequently reach the cells of the epithelium [38]. Considering this, we can expect that a large fraction of mixed micelles formed during in the *in vitro* digestion of these products may be highly bioaccessible, especially in the GP, whose MP is significantly higher. Finally, in both MPs, a certain population of particles can be observed between 500 and 2000 nm, which can be attributed to the presence of micelle aggregates [40].

## 3. Materials and Methods

### 3.1. Materials

A supplement based on a lipid formulation of arachidonic acid and docosahexaenoic acid (Enfamil^®^ DHA & ARA) was supplied by Mead Johnson & Company, LLC (Chicago, IL, USA) and stored at room temperature in the dark. The fat content of this product, determined by the Folch method, is 35.1%. This data, therefore, is used to subsequently calculate the amounts to be added to the reaction medium to carry out the gastrointestinal digestion *in vitro*. AquaCelle^®^, a self-emulsifying product specific for the delivery of lipids and the improvement of their bioaccessibility, was supplied by Pharmako Biotechnologics (Frenchs Forest, Australia) and was stored in the dark at 4 °C. An oil based on ARA and DHA, in a 2:1 ratio, marketed under the name Formulaid^TM^ was supplied by DSM (Heerlen, Limburg, Germany) and was stored in the dark at 4 °C (Table S1). Human milk was donated by the neonatology service of the La Paz university hospital (Madrid, Spain), and was stored at −20 °C. Microalgae oil from the microalgae *Schizochytrium* sp. was supplied by Progress Biotech (Capelle aan den Ijssel, The Netherlands) and was stored under a modified atmosphere at 4 °C (Table S2). Arachidonic acid oil purchased from Penta Manufacturing (West Caldwell, NJ, USA) was stored under a modified atmosphere at 4 °C. The reagents used in the *in vitro* digestion, trizma, maleic acid, pepsin, bile salts (SB), and cholesterol, were supplied by Sigma-Aldrich (St. Louis, MO, USA). Additionally, a sodium chloride solution (NaCl, supplied by Scharlab S.L. (Scharlab S.L., Mas d’En Cisa, Spain)) and a calcium chloride solution (CaCl_2_, supplied by Panreac (Barcelona, Spain)) were used. Rabbit gastric lipase (>15 USP/mg) was supplied by Lipolytech (Marseille, France), egg yolk phosphatidylcholine (PC) was supplied by Lipoid (Ludwigshafen, Germany), and pancreatin from pig pancreas (≥2.0 USP/mg) was supplied by MP Biomedicals, LLC (Irvine, CA, USA) and was stored in a freezer (−20 °C). Food-grade phospholipase A2 (PLA2) from *Streptomyces violaceoruber* (103 U mg^−1^) was supplied by Nagase Chemtex Corporation, Fukuchiyama Factory (Kyoto, Japan) and stored under refrigeration.

Purified acylglycerol standards of microalgae oil and arachidonic acid oil (TAG, DAG, and MAG) were obtained from an enzymatic glycerolysis product by solid phase extraction (SPE) using a Supelco SupelcleanTM LC-Si SPE cartridge, 3 mL, 500 mg (Sigma-Aldrich, St. Louise, fifteen USES). The elution method used was that described by Ingalls et al. [41]. For this, glycerolysis was carried out according to the method described by Corzo-Martínez, et al. [40], for which the enzyme Novozym^®^ 435 (Novozymes, Bagsværd, Denmark) and glycerin (Scharlab S.L., Mas d’En Cisa, Spain) were used. On the other hand, a pure standard of free fatty acids obtained from the mixture in proportion (2:1) of arachidonic acid oil hydrolysis product and microalgae oil hydrolysis product was prepared according to Chabni et al. [36].

The solvents used were chloroform, methanol, hexane, methyl tert-butyl ether (MTBE), petroleum ether, ethanol, isopropanol, ethyl acetate, and acetic acid, all supplied by Macron (Avantor Performance Materiale, Center Valley, PA, USA), and formic acid (98% purity), supplied by Panreac (Barcelona, Spain); all of them were HPLC and GC grade. BF3 (14% in methanol) (Supelco, Pasadena, CA, USA) and anhydrous sodium sulfate (Panreac, Barcelona, Spain) were also used.

### 3.2. Methods

#### 3.2.1. Preparation of the Products to Be Digested

In this work, the gastrointestinal *in vitro* digestion of three products was carried out: Enfamil^®^ DHA & ARA, an emulsion composed of Formulaid^TM^, AquaCelle^®^, and pasteurized donated human milk, and a glycerolysis product. Of these three products, the last two had to be prepared, as follows. To prepare the emulsion of Formulaid^TM^, AquaCelle^®^, and pasteurized donated human milk, the following steps were followed: 4 parts of Formulaid^TM^ were mixed with 1 part of AquaCelle^®^ at room temperature and allowed to rest for 10 min. Then, the mixture was sonicated with the Vibra cell sonicator, VC 130 (Sonics, Newton, MA, USA), for 1 min at 75% power (25 Hz), under nitrogen and in an ice bath. It was allowed to rest for 1 min and then, the donated breast milk was added, in a 1:1 ratio, and sonicated again in the same way. This last step was repeated 3 more times, with intervals of 1 min of rest and 1 min of sonication, so the emulsion would have been sonicated 5 times. Subsequently, it was sonicated in the same way for 2 min, with intervals of 2 min of rest, 2 times in total, so that at the end of the procedure the emulsion would have been sonicated a total of 9 times.

To prepare the glycerolysis product, an enzymatic glycerolysis was carried out according to the method described by Corzo et al. [11]. For this, arachidonic acid oil and microalgae oil were mixed in a 2:1 ratio in a 120 mL vial, and 10% (*w*/*w*) of glycerin was added. The mixture was shaken in the IKA^®^ KS 4000 ic control orbital (IKA, Staufen, Germany) at 40 °C and 200 rpm for 10 min. Next, 1 g of the Novozym^®^ 435 enzyme was added, and it was left stirring in an orbital in the dark, at 40 °C and 200 rpm for 48 h. After this time, it was filtered In a Kitasato with a hot ceramic filter and the product obtained was stored in a nitrogen atmosphere in a refrigerator.

#### 3.2.2. *In Vitro* Gastrointestinal Digestion Model

The *in vitro* gastrointestinal digestion of the three products (Enfamil^®^ DHA & ARA, an emulsion composed of Formulaid^TM^, AquaCelle^®^, and pasteurized donated human milk, and a glycerolysis product) was carried out according to the procedure by Chabni et al. [11]. This methodology comprises two stages: (i) gastric and (ii) intestinal digestion. Briefly, in gastric digestion, fat samples were emulsified with simulated gastric fluid and digested at 37 °C and 900 rpm for 60 min, using gastric lipase and pepsin. In the intestinal phase, pH was adjusted to 7.5, a simulated mixture of bile secretion and enzymes (pancreatin and phospholipase A2) was added, and digestion was carried out at 37 °C and 900 rpm for 60 min. Aliquots at different times were periodically withdrawn from either gastric (400 µL) or intestinal (1 mL) digestion medium to be subsequently analyzed. The final digestion product was centrifuged at 4500 rpm and 37 °C for 45 min (Thermo Scientific Sorvall 6000 LYNX, Thermo Fisher Scientific, Waltham, MA, USA) according to the method described by Corzo-Martínez et al. [40]. Three phases were obtained: an upper oily phase (OP), formed by the undigested lipid fraction; an intermediate aqueous fraction, called the micellar phase (MP), which contains the lipid fraction digested in the form of micellar and vesicular structures, and a precipitate phase (PP), which consists of a lower phase containing insoluble compounds at 37 °C. These phases were separated for the subsequent extraction of the lipids present in each of them. Lipid extraction was performed according to the methodology of Chabni et al. [11], and subsequently samples were analyzed by GC.

#### 3.2.3. Lipid Identification and Quantification by Gas Chromatography

Lipid samples obtained from gastrointestinal digestion were analyzed by gas chromatography (GC). GC consisted of an Agilent 7820A gas chromatograph (Agilent Technologies, Santa Clara, CA, USA) with on-column injection coupled to a flame ionization detector (FID). The chromatographic separation was based on the method developed by Torres et al. [42]. An HP-5MS capillary column (7 m × 0.25 mm internal diameter × 0.25 μm) was used. The injection volume was 0.2 μL. The temperatures of injector and detector were 50 and 340 °C, respectively. The program of temperatures started at 60 °C, increasing at 42 °C min^−1^ until 250 °C. This temperature was maintained for 20 min and then increased up to 340 °C at 25 °C min^−1^, which was maintained for 20 min. The flow rate of the carrier gas (helium) was maintained at 1 mL min^−1^.

The quantification was performed by the external standard method using pure standards. For this, calibration curves were made with each of the standards. These data are expressed as the percentage of TAGs, DAGs, MAGs, and FFAs with respect to the weight of the residue (w.r.) obtained after lipid extraction, according to the following equation:

(1) WcompWw.r.×100

where W_comp_ is the weight of each type of lipid and W_w.r._ is the weight of the residue obtained after lipid extraction.

The distribution of each lipid type was determined by combining the relative weight of each phase and composition according to the following equation:

% of A = weight percent of oil phase × (percentage of A in oil phase/100) + weight percentage of the micellar phase × (percentage of A in the micellar phase/100) + weight percentage of the precipitated phase × (percentage of A in the precipitated phase/100)(2)

where A refers to any of the types of lipids analyzed in this study.

#### 3.2.4. Physiochemical Characterization: Particle Size Distribution and Electrical Charge of the Micellar Structures

The particle size distribution and ζ-potential of the micellar phases were measured by light scattering by phase analysis (Zetasizer Nano-ZS, Malvern Instruments (Westborough, MA, USA). The instrument was equipped with a 632.8 nm wavelength He-Ne laser. Measurements were performed at a scattering angle of 173° at a temperature of 25 °C. The micellar phase of the samples was diluted 1:10 (*v*:*v*) to perform the analysis [39]. These analyses were performed in triplicate.

#### 3.2.5. Data Analysis

All determinations were performed in triplicate. The data were analyzed in terms of the variance (ANOVA). The ANOVA and regression analyses were performed according to the MStatC (1.12 version) and SlideWrite (4.0 version) software. Duncan’s multiple range test was employed to determine significant differences between the means. *p* values < 0.05 were considered statistically significant.

## 4. Conclusions

The digestibility of the FormulaidTM, AquaCelle®, and pasteurized donated human milk emulsion, and the commercial Enfamil® DHA & ARA emulsion was found to be suboptimal. Large oily phases were obtained after the centrifugation of both digestion products. Additionally, a limited amount of monoacylglycerols was produced after *in vitro* digestion in these two emulsions. These findings indicate reduced digestibility and subpar bio-accessibility.

In contrast, the self-emulsifying lipid obtained via enzymatic glycerolysis demonstrated significantly better bio-accessibility, with less than 14% oily phase after the centrifugation of the digestion product. Furthermore, approximately 17% monoacylglycerols and a micellar phase exceeding 80% were obtained. Additionally, more than 40% of diacylglycerols in the digestion product were located in the micellar phase, indicating that this digestion product exhibits strong emulsification properties and is highly suitable for the administration of ARA and DHA in preterm infants.

The superior bioaccessibility of the glycerolysis product suggests its potential application in preterm infant nutrition. Future research could explore the in vivo validation of its efficacy and evaluate the economic feasibility of implementing this formulation at a larger scale.

## Figures and Tables

**Table 1:** Parameters associated with the rate of hydrolysis for the three formulations studied.

Product	Intercept	Slope	Adj. R^2^
Formulaid^TM^, AquaCelle^®^, and donated breast milk emulsion	0.054 ± 0.044	−0.010 ± 0.003	0.760
Enfamil^®^ DHA & ARA	0.203 ± 0.100	−0.028 ± 0.006	0.842
Enzymatic glycerolysis product	0.071 ± 0.017	−0.022 ± 0.001	0.992

**Table 2:** ζ-potential of the glycerolysis product and commercial Enfamil^®^ DHA & ARA.

Product	ζ-Potential
Enfamil^®^ DHA & ARA	−41.5 ± 5.6
Enzymatic glycerolysis product	−42.9 ± 2.2

## Data Availability

The data presented in this study are available in the article.

## Supplementary Materials

The following supporting information can be downloaded at: https://www.mdpi.com/article/10.3390/molecules29246032/s1, Table S1: Formulaid™ (2:1) specifications; Table S2: Microalgae oil composition.

## Abbreviations

ARA: arachidonic acid. DHA: docosahexaenoic acid. GP: glycerolysis product. OP: oil phase. MP: micellar phase. PP: precipitate phase. TAG: triacylglyceride. DAG: diacylglyceride. MAG: monoacylglyceride. FFA: free fatty acids. NEFA: non-esterified fatty acids. LCPUFAs: long-chain polyunsaturated fatty acids. ζ-potential: zeta potential.
