# Supplementary material for: A Comparative In Vitro Digestion Study of Three Lipid Delivery Systems for Arachidonic and Docosahexaenoic Acids Intended to Be Used for Preterm Infants
Source: Molecules. 2024 Dec 21;29(24):6032. doi: 10.3390/molecules29246032 (PMC11679688; doi:10.3390/molecules29246032)
Supplement: Supplementary file 1 [file molecules-29-06032-s001.zip › molecules-3306315-supplementary.pdf]

Supplementary Material

Table S1. Formulaid™ (2:1) specifications

Appearance	clear free flowing liquid at 40°C
Color	light yellow to orange
Aroma	characteristic
Arachidonic acid, mg/g content	240 – 290 mg/g
Docosahexaenoic acid, mg/g content	120 – 150 mg/g
Peroxide value	max. 5.0 meq/kg
Anisidine value	max. 20
Free fatty acid	max. 0.4 %
Unsaponifiable matter	max. 3.5 %
Trans fatty acids	max. 1 %
Arsenic	Arsenic: max. 0.1 ppm
Cadmium	Cadmium: max. 0.1 ppm
Copper	Copper: max. 0.1 ppm
Iron	Iron: max. 0.2 ppm
Lead	Lead: max. 0.1 ppm
Mercury	Mercury: max. 0.04 ppm
Phosphorus	Phosphorus: max. 10 ppm
**Fatty acid profile, area percent**	
10:0	max. 1.0 %
12:0	max. 2.0 %
14:0	3.0 – 8.0 %
16:0	5.0 – 17.0 %
16:1	max. 2.0 %
18:0	3.0 – 14.0 %
18:1	6.0 – 36.0 %
18:2	3.0 – 12.0 %
18:3	max. 7.0 %
20:0	max. 1.0 %
20:3	max. 7.0 %
20:4	26.0 – 30.0 %
22:0	max. 2.0 %
22:6	13.0 – 15.0 %
24:0	max. 2.0 %
24:1	max. 1.0 %
Others	max. 3.0 %

Table S2. Microalgae oil composition

	Specification
Schizochytrium sp. (WZU477) oil	Description/Definition:<br>The novel food is an oil produced from the strain WZU477 of the microalgae Schizochytrium sp.<br><br>Composition:<br>Acid value: ≤ 0,5 mg KOH/g Peroxide value (PV): ≤ 5,0 meq/kg oil Moisture and volatiles: ≤ 0,05 % Unsaponifiables: ≤ 4,5 % Trans-fatty acids: ≤ 1,0 % Docosahexaenoic acid (DHA): ≥ 32,0 % p-anisidine value: ≤ 10′
